# Genetic Diversity and Population Structure Analysis of *Pinus elliottii* Germplasm Resources in Jiangxi Province

**DOI:** 10.3390/life14111401

**Published:** 2024-10-31

**Authors:** Min Yi, Rong Hu, Wending Huang, Tingxuan Chen, Wenlei Xie, Haiping Xie, Xin Luo, Meng Lai

**Affiliations:** Jiangxi Provincial Key Laboratory of Subtropical Forest Resources Cultivation, 2011 Collaboration Innovation Center of Jiangxi Typical Trees Cultivation and Utilization, College of Forestry, Jiangxi Agricultural University, Nanchang 330045, China; yimin6104@163.com (M.Y.); hurong089@163.com (R.H.); hwd0208@163.com (W.H.); sea1067488960@163.com (T.C.); xwl981222@163.com (W.X.); 13302512932@163.com (H.X.); 18942335817@163.com (X.L.)

**Keywords:** *Pinus elliottii*, SSR markers, germplasm resources, genetic diversity, genetic differentiation, breeding strategies

## Abstract

This study aimed to compare and assess the genetic diversity and trends among the introduced family provenance, first-cycle superior trees breeding provenance, and improved-generation superior trees breeding provenance of *Pinus elliottii* using EST-SSR markers. The goal was to provide a foundation for advanced genetic improvement and sustainable utilization of *P. elliottii* in Jiangxi Province. A total of 417 individuals were analyzed for their genetic diversity and population structure using 19 pairs of SSR markers. The analysis identified 103 alleles across all the samples, with an average of 5.421 alleles per locus. Compared to other coniferous species, *P. elliottii* exhibited a moderate to high level of genetic diversity (*I* = 0.862, *He* = 0.457). Analysis of the molecular variance (AMOVA) revealed that 97.90% of the genetic variation occurred within provenances, consistent with a low genetic differentiation coefficient (*Fst* = 0.016 < 0.05) and high gene flow (*Nm* = 15.715) among provenances. In addition, analysis using STRUCTURE v. 2.3.4 software divided the 417 germplasm samples into two distinct groups, corroborating the results of the principal coordinates analysis (PCoA) and the unweighted pair group method with arithmetic (UPGMA) clustering analysis. Overall, the germplasm resources of *P. elliottii* exhibited rich genetic diversity, with the majority of the genetic variation occurring within provenances. For the genetic improvement of high-resin-yielding slash pines, breeding programs should prioritize populations with high genetic diversity while carefully selecting superior individuals from within those populations. These findings provide a solid foundation for breeding high-resin-yielding varieties and for future research on the sustainable utilization of these valuable resources.

## 1. Introduction

*Pinus elliottii*, native to the southeastern United States, is a valuable species for timber and pulp production, as well as a high-quality source of resin [1,2]. The resin from *P. elliottii* is renowned for its high yield, excellent quality, resistance to crystallization, slow coagulation, and low impurity content. Since its introduction into Jiangxi Province in 1948, *P. elliottii* has become a key afforestation species in the hilly areas of the Jitai Plain, primarily due to its strong adaptability, rapid growth, and high resin yield [3]. To accelerate the selection and breeding of high-resin-yielding *P. elliottii* varieties, genetic improvement efforts began in China in the 1980s. These initiatives have focused on genetic variation in resin-related traits, resin composition, and molecular breeding for resin production, laying the groundwork for advanced genetic improvement of *P. elliottii* [4,5,6]. Interspecific hybridization is a vital approach for germplasm innovation in pine trees, expanding genetic resources and incorporating favorable traits from both closely related and distantly related species. This strategy addresses the limitations of close-relative hybridization by broadening the gene pool [7]. Previous research has explored the cross-compatibility of *P. elliottii* with native Chinese pine species (*Pinus massoniana*) and exotic species (*Pinus taeda* and *Pinus caribaea*). The results indicated that three hybrid combinations—*Pinus caribaea* × *P. elliottii*, *P. elliottii* × *Pinus caribaea*, and *P. elliottii* × *Pinus taeda*—exhibited fertility levels equal to or higher than their corresponding half-sib controls. In contrast, the *Pinus massoniana* × *P. elliottii* and *Pinus taeda* × *P. elliottii* hybrids showed significantly lower fertility than the half-sib controls, with some exhibiting little to no fertility [8]. Furthermore, *P. elliottii* is typically characterized by dense foliage and large lateral branches during its juvenile phase, contributing to the severe damage during the 2008 snow disaster in southern China. Therefore, hybridization to improve the needle size and lateral branch traits holds significant potential for enhancing its resilience to such events [9,10].

However, due to the lack of natural *P. elliottii* germplasm resources in China, the genetic foundation for breeding improvements depends heavily on the diversity of the introduced germplasm. Early introductions were often driven by specific objectives, with limited consideration of population genetic diversity, which has limited the efficiency and effectiveness of *P. elliottii* breeding programs. As a result, a comprehensive study of the genetic base of breeding resources in Jiangxi Province is crucial to better understand the genetic information and background of the germplasm. This will facilitate the development of breeding populations with enhanced genetic quality and provide essential theoretical guidance for future genetic improvement and the creation of *P. elliottii* germplasm. Foundational studies on the genetic diversity of breeding populations have been conducted for many conifer species, both domestically and internationally, including Masson pine (*Pinus massoniana*) [11], Scots pine (*Pinus sylvestris*) [12], loblolly pine (*Pinus taeda*) [13], Korean pine (*Pinus koraiensis*) [14], and European larch (*Larix decidua*) [15].

Genetic diversity can be assessed through a variety of approaches, including morphological, cytological, physiological, biochemical, and molecular markers [16]. Early studies primarily used observable morphological traits to analyze genetic variation, focusing on phenotypic evaluation. However, morphological markers are limited in number, lack standardized observation criteria, and are highly influenced by environmental factors, making them less suitable for genetic variation studies [17]. In contrast, molecular markers represent the genetic diversity at the DNA level, offering greater precision as they are unaffected by environmental conditions [18]. Among the available molecular markers, simple sequence repeat (SSR) markers are particularly favored due to their abundance, high reliability, and convenient operation [19,20]. SSR markers have been widely applied in studies of genetic diversity, population structure, and phylogenetic relationships in forest tree germplasm, such as Masson pine [11], Scots pine [21], Chinese pine (*Pinus tabuliformis*) [22], and Korean pine [14]. Although SSR analysis of *P. elliottii* has been employed in studies such as transcriptomes [6,23], SSR marker development [24], genetic diversity studies using RAPD molecular markers in seed orchards [25], exome resequencing [26], and genetic map construction [27], comprehensive studies on the genetic diversity of *P. elliottii* germplasm resources remain limited.

Although our research group has collected a substantial amount of *P. elliottii* germplasm from both the United States and various regions across China, the genetic background of these diverse sources remains unclear. This has led to issues, such as germplasm admixture, genetic background ambiguity, and a lack of systematic integrated research, making it difficult to select suitable breeding materials. Therefore, assessing the genetic background of these populations is crucial. To ensure a robust genetic foundation for the sustainable improvement of *P. elliottii*, this study focused on the analysis of the genetic diversity and population structure across breeding provenances with different levels of improvement, using SSR molecular markers. The materials included the introduced family provenance, first-cycle superior trees breeding provenance, and improved-generation superior trees breeding provenance collected from Jiangxi Province. The aim was to elucidate the complexity of their genetic backgrounds, the relationships among materials, and the degree of genetic variation. This research provides a theoretical basis and material foundation for parent selection in hybrid breeding, as well as for constructing core germplasm and fingerprinting profiles. Moreover, it is expected to improve the efficiency of breeding high-resin-yielding *P. elliottii* varieties while laying the groundwork for further exploration and utilization of high-resin-yielding germplasm in the future.

## 2. Materials and Methods

### 2.1. Experimental Materials

The experimental materials for this study consisted of 417 *P. elliottii* germplasm resources currently preserved in Jiangxi Province. Samples were collected from young buds or leaves, which were immediately flash-frozen in liquid nitrogen and stored at −80 °C until further use. The primary sources of these samples are detailed below (Table 1).

Introduced family provenance (IP): This group includes 113 families from the *P. elliottii* provenance/family trial forest established in 1990 at Baiyunshan Forest Farm (27.22° N, 115.13° E), Qingyuan District, Ji’an City. The seeds were sourced from seed orchards in Georgia, Mississippi, and Florida, USA, with a total of 112 samples successfully amplified. Additionally, a family trial forest established in 2011 and 2018 at Fengshushan Forest Farm (29.37° N, 117.25° E), Jingdezhen, includes 30 and 10 families, respectively, also sourced from seed orchards in Florida, USA, with 18 samples successfully amplified. Furthermore, the superior family trial forest established in 2018 by the Ganzhou Forestry Research Institute (25.38° N, 114.93° E) includes 40 families sourced from seed orchards in Arkansas, USA, with all 40 samples successfully amplified.

First-cycle superior trees breeding provenance (FP): This group primarily consists of slash pine plantation stands from early introductions in Jiangxi Province. A total of 44 samples were collected from the first-generation seed orchard established in May 1992 at Baiyunshan Forest Farm, Qingyuan District, Ji’an City. An additional 77 samples were collected from the first-generation seed orchard established in 1980 at Xiajiang County Superior Tree Breeding Farm (27.58° N, 115.32° E).

Improved-generation superior trees breeding provenance (IGP): This group includes 126 samples from experimental forests planted with selected superior trees from across the province, with 79 samples from Baiyunshan Forest Farm, Qingyuan District, Ji’an City, and 47 samples from Xiajiang County Superior Tree Breeding Farm.

### 2.2. Research Methods

#### 2.2.1. Genomic DNA Extraction and Detection

Genomic DNA was extracted using the DNAsecure Plant Kit (DP320) from Tiangen Biotech Co., Ltd. (Beijing, China). The concentration and quality of the extracted DNA were assessed via 1% agarose gel electrophoresis. The DNA samples were then diluted to a uniform concentration of 20–50 ng/μL and stored at −20 °C for further analysis.

#### 2.2.2. SSR Primer Screening

A total of 24 pairs of EST-SSR primers were selected from the published literature [24]. Additionally, SSR loci were identified within unigenes obtained from the *P. elliottii* transcriptome using MISA software (http://misaweb.ipk-gatersleben.de/, accessed on 25 October 2024), with the following criteria: dinucleotide repeats > 6 times, and trinucleotide, tetranucleotide, pentanucleotide, and hexanucleotide repeats > 5 times. Based on the conserved regions flanking the SSR loci, 103 pairs of SSR primers were designed using Primer 3.0 software [28] and synthesized by Sangon Biotech Co., Ltd. (Shanghai, China). The primers were validated for polymorphism using 11 slash pine samples.

#### 2.2.3. PCR Amplification and Capillary Electrophoresis

The PCR reaction system and program are detailed in Table 2. Primers that produced clear and polymorphic amplification bands were labeled with one of four fluorescent dyes (FAM, Rox, Tem, or Hex). The PCR products were then analyzed using capillary electrophoresis on an ABI 3730XL DNA sequencer in collaboration with Sangon Biotech Co., Ltd. (Shanghai, China).

#### 2.2.4. Statistical Analysis

Microsatellite allele data for each *P. elliottii* sample, obtained from capillary electrophoresis, were analyzed using GeneMarker v.2.2.0 software [29]. DataFormater v.2.7 [30] and GenAlEx v.6.5 software [31] were then used to convert the resulting data into the necessary formats for further analysis.

The genetic diversity parameters, including the number of alleles (*Na*), the effective number of alleles (*Ne*), Shannon’s information index (*I*), observed (*Ho*) and expected heterozygosity (*He*), F-statistics (*Fis*, *Fit* and *Fst*) and gene flow (*Nm*), were calculated using POPGENE v.1.3.1 software [32]. The allelic frequency variation and polymorphic information content (PIC) were assessed and calculated using PowerMarker v.3.25 software [33].

To assess the degree of genetic variation within and between the three *P. elliottii* provenances, an analysis of molecular variance (AMOVA) was performed using ARLEQUIN v.3.5.2.2 software [34].

The genetic structure of the 417 individuals was analyzed using STRUCTURE v.2.3.4 software [35]. The number of clusters (K) was set from 1 to 10, with a burn-in period of 100,000 iterations and 10,000 MCMC repetitions. Each K value was run three times, and the optimal number of clusters was determined by plotting K against ∆K and selecting the peak value.

The genetic distances between samples or provenances were calculated using PowerMarker v.3.25 software, and a UPGMA dendrogram was constructed based on these distances using MEGA v.11.0 software [36]. In addition, principal coordinate analysis (PCoA) was conducted using GenAlEx v.6.5 software to further explore the genetic relationships between provenances based on the microsatellite allele data.

## 3. Results

### 3.1. Polymorphism Analysis of EST-SSR Markers

**Figure 1:**
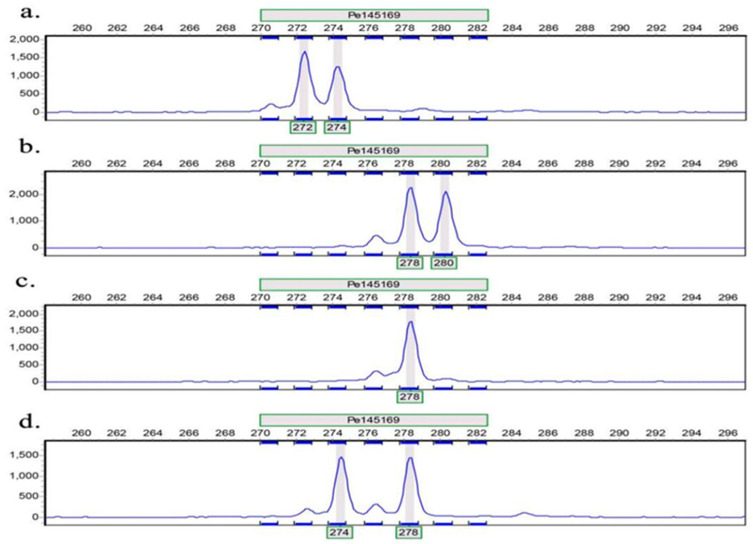
Amplification of some samples on primer Pe145169. The horizontal axis represents the size of the product (bp); The vertical axis represents the relative fluorescence signal; (**a**–**d**) represent four different samples.

The capillary electrophoresis results showed that out of the 127 primer pairs tested, 112 successfully amplified bands, yielding an amplification success rate of 88.19%. Among these, 19 primer pairs were identified as highly polymorphic SSR markers, with a polymorphic loci ratio of 14.96% (Figure 1; Table 3).

### 3.2. Cross-Species Applicability of EST-SSR Markers

To evaluate the cross-species applicability of the 19 polymorphic SSR markers developed for *P. elliottii*, 15 samples each of *Pinus massoniana* and *Pinus taeda* were selected for validation. The results (Table 4) demonstrated that all 19 SSR markers successfully amplified bands in both species, achieving a cross-species amplification rate of 100%.

### 3.3. Genetic Diversity Analysis of Loci

A total of 103 alleles were detected across the 417 *P. elliottii* samples using the 19 pairs of SSR primers, with an average of 5.421 alleles per locus (Table 5). Primer Pe103802 exhibited the highest number of alleles (13), with an effective number of alleles of 1.777, followed by primer Pe113019, which had 8 alleles and an effective allele number of 1.263. Primers Pe123077 and Pe139538 had the fewest alleles, with two each, and effective allele numbers of 1.964 and 1.352, respectively. Shannon’s information index (*I*) ranged from 0.370 (Pe114582) to 1.638 (Pe145169), with an average value of 0.862. The observed heterozygosity (*Ho*) ranged from 0.138 (Pe114582) to 0.795 (Pe123077), while the expected heterozygosity (*He*) ranged from 0.149 (Pe114582) to 0.777 (Pe145169), with average values of 0.402 and 0.457, respectively. For most loci, *He* was greater than *Ho*, except for primers Pe119033, Pe106732, and Pe123077, indicating varying degrees of heterozygote deficiency. The polymorphism information content (PIC) values ranged from 0.146 (Pe114582) to 0.743 (Pe145169), with an average of 0.411. Among the loci, 3 had PIC values below 0.25, indicating low polymorphism; 10 loci had PIC values between 0.25 and 0.5, indicating moderate polymorphism; and 6 loci had PIC values greater than 0.5, indicating high polymorphism. Overall, these 19 SSR markers proved highly effective for analyzing the genetic diversity of the *P. elliottii* provenances.

### 3.4. Comparison of Genetic Diversity Among Provenances

A total of 83 and 95 alleles were detected in the FP and IGP of *P. elliottii*. In contrast, only 82 alleles were found in the introduced family provenance (Table 6). The ratios of the effective number of alleles (*Ne*) to the total number of alleles (*Na*) were higher in both the FP and IGP compared to the IP. Notably, the IP had a higher number of alleles at locus 135178 than the other two provenances. The FP exhibited the highest number of alleles at loci 144426 and 131259, while the IGP showed the highest number of alleles at seven loci: 145380, 119033, 114582, 138370, 134815, 113019, and 103802.

The Shannon’s information index (*I*) values for the IP, FP, and IGP were 0.734, 0.839, and 0.964, respectively. The PIC values were 0.356, 0.407, and 0.460, respectively. The average *Ho* values were 0.371, 0.421, and 0.424, while the average *He* values were 0.398, 0.457, and 0.512, respectively. Overall, the genetic diversity of the FP and IGP was higher than that of the IP. These findings suggest that the *P. elliottii* provenances have maintained a relatively high level of genetic diversity following selective breeding and improvement efforts.

### 3.5. Genetic Differentiation and Gene Flow Among Provenances

As shown in Table 7, the *Fis* values across the 19 SSR loci ranged from −0.655 (Pe123077) to −0.401 (Pe139538), with an average of 0.110, indicating the presence of heterozygote deficiency within the provenances. The average *Ho* (0.402) was lower than the average *He* (0.457), further confirming the occurrence of heterozygote deficiency. The *Fit* values ranged from −0.634 (Pe123077) to −0.451 (Pe139538), with an average of 0.124. The overall *Fst* values ranged from 0.005 (Pe145380) to 0.084 (Pe139538), with an average of 0.016, indicating low genetic differentiation among the *P. elliottii* provenances. The gene flow (*Nm*) values ranged from 2.710 (Pe139538) to 53.260 (Pe145380), with an average of 15.715, suggesting substantial gene flow between the provenances, which likely reduces the genetic drift and contributes to the observed low genetic differentiation.

The AMOVA analysis based on the 19 loci (Table 8) revealed that the genetic variation among the provenances accounted for only 2.10% of the total variation, while 97.90% of the genetic variation was attributable to differences within individuals and among individuals within provenances. This indicated that most of the genetic variation in *P. elliottii* occurred within individuals, rather than between the IP, FP, and IGP.

The genetic similarity coefficients and genetic distances among the three provenances were calculated using Nei’s method (Table 9). The genetic distance between the IP and the FP was 0.014, with a genetic similarity coefficient of 0.986. The genetic distance between the IP and the IGP was 0.020, with a genetic similarity coefficient of 0.980. The genetic distance between the FP and the IGP was 0.019, with a genetic similarity coefficient of 0.981. These results indicate that the IP is more closely related to the FP than to the IGP.

### 3.6. Population Genetic Structure Analysis

**Figure 2:**
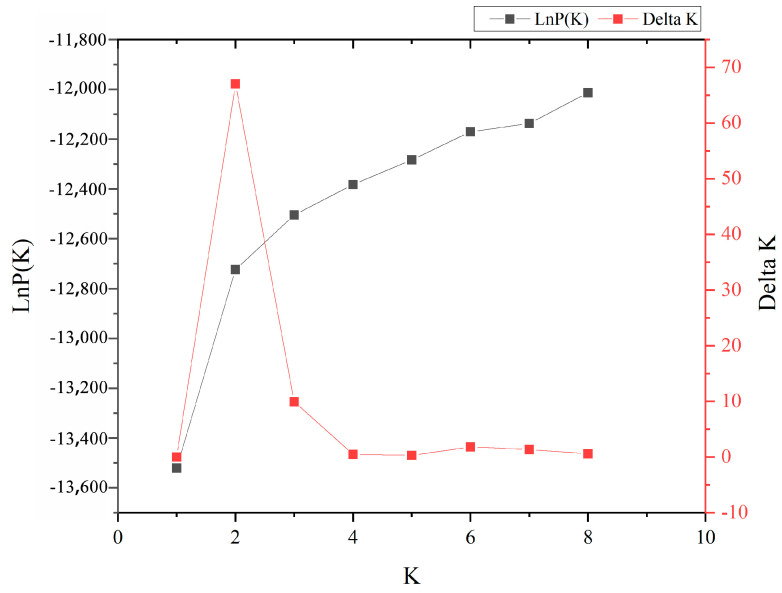
Diagnostic plots of the LnP(D) and ΔK from the STRUCTURE analysis of the *P. elliottii* provenances.

**Figure 3:**
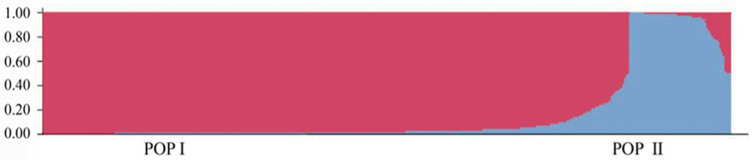
Results of the structure analysis of the *P. elliottii* provenances when K = 2. Red represents Subgroup I, blue represents Subgroup II.

To further investigate the population genetic structure and relationships among the 417 *P. elliottii* samples, STRUCTURE software was used to analyze the data based on 19 pairs of SSR primers. The results (Figure 2) showed that as the K value increased, the lnP(D) also increased continuously. The most significant change in the lnP(D) occurred when the K value increased from 1 to 2, with the ∆K reaching its maximum peak at K = 2, indicating that the 417 *P. elliottii* samples were divided into two distinct subgroups (Figure 3).

To better characterize the genetic background of the materials, a Q value > 0.6 was used as the threshold for assigning the 417 *P. elliottii* samples into different subgroups. Based on this criterion, 410 samples were assigned to either Subgroup I or Subgroup II, indicating the relatively homogeneous genetic backgrounds among these samples. The 417 samples originated from the IP, FP and IGP. However, they were not strictly grouped according to their original provenances. Thus, the origins of the materials within each subgroup were further analyzed (Table 10). Subgroup I comprised 84.41% of the total samples, making it the most abundant group, with 352 samples derived from the introduced family trial forest (159 samples), the first-generation seed orchard (105 samples), and the advanced-generation seed orchard (88 samples). Subgroup II included 58 samples, with 4 from the introduced family trial forest, 16 from the first-generation seed orchard, and 38 from the advanced-generation seed orchard. Additionally, seven samples from the introduced family trial forest were placed in a mixed group, as they displayed mixed ancestry and complex genetic structures. These findings suggest that caution should be exercised when selecting these samples as breeding parents.

### 3.7. PCoA and Genetic Clustering

**Figure 4:**
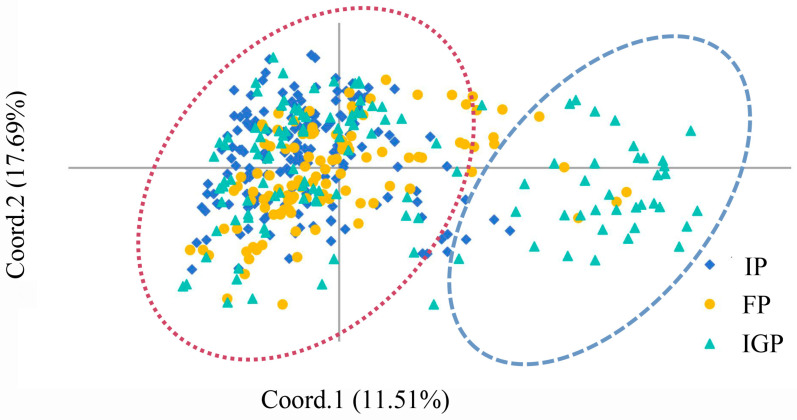
Principal coordinates analysis based on Nei’s genetic distance of *P. elliottii*.

To further analyze the cluster patterns, PCoA of *P. elliottii* was performed based on the pairwise genetic distance matrix of 19 EST-SSRs, with the results shown in Figure 4. The analysis revealed that the first principal coordinate (Coord. 1) and second principal coordinate (Coord. 2) accounted for 11.51% and 17.69% of the genetic variation, respectively. The individuals from the IP, FP, and IGP could not be completely separated, suggesting relatively close genetic relationships among these provenances. The entire *P. elliottii* germplasm could be roughly divided into two groups, which is consistent with the results of the STRUCTURE analysis at K = 2.

**Figure 5:**
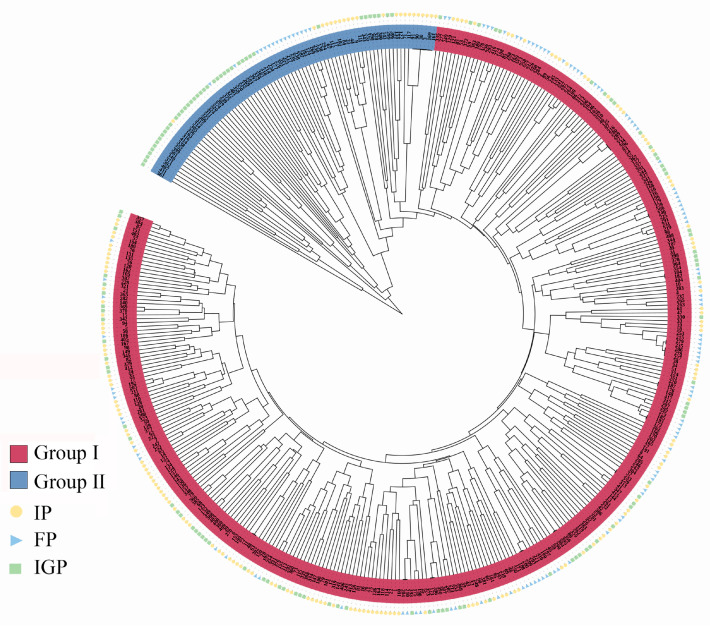
UPGMA cluster analysis of the *P. elliottii* provenances based on the genetic distance.

A clustering dendrogram of the 417 *P. elliottii* germplasm samples was constructed using the unweighted pair group method with arithmetic mean (UPGMA) in MEGA software. As indicated by the clustering results (Figure 5), the *P. elliottii* germplasm did not cluster strictly according to the IP, FP, and IGP. Instead, the samples were broadly divided into two groups. Except for a small number of samples with discrepancies, the clustering results were largely consistent with the findings from the PCoA and STRUCTURE analyses.

## 4. Discussion

### 4.1. Development and Cross-Species Applicability of P. elliottii Primers

The limited availability of SSR primers has hindered molecular-level research on *P. elliottii* populations. In this study, we developed 19 polymorphic SSR loci based on the *P. elliottii* transcriptome and conducted cross-species applicability analyses across three pine species. The results demonstrated that the SSR markers developed for *P. elliottii* exhibited high cross-species applicability in other *Pinus* species, such as *P. taeda* and *P. massoniana*, with a 100% amplification rate and a high proportion of polymorphic loci—89.47% in *P. taeda* and 100% in *P. massoniana*. This high level of cross-species applicability is likely due to the conserved nature of the EST sequences compared to the non-coding DNA, along with the low molecular evolution rates within the *Pinus* genus, which facilitate the cross-transferability of SSRs and other molecular markers [37,38]. For instance, 14 EST-SSR markers developed for Aleppo pine (*Pinus halepensis*) showed high applicability in Mediterranean pines (*Pinus halepensis*) [39], and 184 EST-SSR markers developed from the lodgepole pine transcriptome were effectively amplified across different Pinus species, with amplification rates ranging from 54% to 73%, including a 65% amplification rate in *P. elliottii* [40]. Similarly, 20 EST-SSR markers developed for *Larix principis-rupprechtii* produced clear and stable bands across three related *Larix* species [41]. In this study, the SSR primers developed for *P. elliottii* demonstrated 100% cross-species applicability and a high proportion of polymorphic loci in *P. massoniana*, consistent with the findings from previous studies. These EST-SSR markers can also be employed in the future for interspecific hybrid identification, comparative mapping, and phylogenetic relationship evaluation among *P. elliottii* and its related species.

The PIC is a crucial genetic parameter often used to assess the discriminatory power of primers and the reliability of the information they provide in genetic diversity studies [42]. According to the definition of polymorphism by Botstein et al., the SSR primers selected in this study had an average PIC value of 0.411, with six loci exhibiting PIC values greater than 0.5, indicating a high level of polymorphism [43]. This suggests that the 19 SSR primers exhibit good polymorphism, effectively reflecting the genetic diversity of the materials. Therefore, these primers can provide technical support for the identification of *P. elliottii* germplasm, variety breeding, and the construction of genetic fingerprints. In comparison to previously developed SSR markers from our research group (average PIC = 0.349), the polymorphism of the primers used in this study was improved and the number of analyzed populations was expanded.

### 4.2. Genetic Diversity of P. elliottii Populations

Genetic diversity is a crucial factor in assessing a species’ ability to adapt to environmental changes. Enhancing the genetic base of a population can increase its adaptability and resilience, thereby improving its overall survival potential [17]. The level of genetic diversity within populations can be reflected by the *He* and the *I* [44].

In this study, 19 pairs of SSR primers were used to amplify three *P. elliottii* provenances, resulting in *I* = 0.862 and *He* = 0.457. These values were higher than those reported for Scots pine (*He* = 0.167; *I* = 0.253) [45], *P. yunnanensis* var. *tenuifolia* (*He* = 0.342; *I* = 0.488) [46], and *P. taiwanensis* Hayata var. *damingshanensis* (*He* = 0.40; *I* = 0.62) [47], but lower than those for *P. massoniana* (*He* = 0.5438; *I* = 0.8696) [11], Chinese pine (*He* = 0.604; *I* = 1.230) [48], and Korean pine (*He* = 0.521; *I* = 1.019) [49]. These results indicate that *P. elliottii* exhibits a moderate to high level of genetic diversity compared to other conifer species.

Some studies suggest that while breeding practices can effectively increase the genetic gains of improved populations, they may also reduce the genetic diversity of these populations due to intensified human selection pressure [50]. However, empirical research has shown that the impact of artificial selection on genetic diversity is not significant. For instance, comparative analyses of the genetic diversity between improved populations obtained through phenotypic selection and their natural source populations in white spruce (*Picea glauca*) found only a slight, non-significant reduction in diversity in the improved populations [51]. Similarly, genetic diversity analyses of first- and second-generation improved seed orchards of *Pinus taeda* and first- to third-generation improved seed orchards of *Larix principis-rupprechtii* revealed that the genetic diversity remained relatively stable across different breeding generations, with only minor population differentiation [52,53]. Even in the fourth-generation breeding population of *Cunninghamia lanceolata,* high genetic diversity was maintained [54], and studies on *Picea sitchensis* [55], *Pinus taeda* [56], and *Larix kaempferi* [50] have found that the genetic diversity of improved populations can sometimes exceed that of their source populations.

In this study, no significant differences were found in the *I*, *Ho*, and *He* among the three provenances of slash pine. However, the genetic diversity of the FP and IGP was slightly higher than that of the IP. These findings align with the results observed in *Larix kaempferi* [50], *Picea sitchensis* [55], and *Pinus taeda* [56], suggesting that the breeding strategies and measures applied during the genetic improvement of slash pine in Jiangxi have been relatively effective. As an introduced species without natural germplasm resources in China, the genetic diversity of slash pine relies on the diversity of the introduced germplasm. To further enhance the genetic diversity of breeding populations, it may be advantageous to introduce superior individuals from native populations with distinct genetic backgrounds or to select elite offspring from already introduced families. This approach would help to further broaden the genetic base of the breeding populations.

### 4.3. Genetic Differentiation and Gene Flow in P. elliottii Populations

Understanding the genetic differentiation and structure of populations is fundamental for developing species conservation strategies and constructing plant breeding populations. Research suggests that a *Fst* value of less than 0.05 indicates low genetic differentiation, 0.05 < *Fst* < 0.15 indicates moderate differentiation, 0.15 < *Fst* < 0.25 indicates relatively high differentiation, and *Fst* > 0.25 indicates high genetic differentiation [57]. In this study, the *Fst* value for *P. elliottii* was 0.016, which is less than 0.05, indicating low genetic differentiation among the populations. Additionally, the AMOVA analysis showed that the within-provenance variation (97.9%) was much greater than the between-provenance variation (2.10%). This, combined with the genetic differentiation analysis, demonstrates that *P. elliottii* has significantly higher genetic diversity within populations than between them, suggesting that selection efforts should focus more on individual trees within populations. This phenomenon has also been observed in other conifer species, such as Korean pine [14], *P. massoniana* [11], Norway spruce [58], *Pinus yunnanensis* var. *tenuifolia* [46], and *Cunninghamia lanceolata* [59]. The likely cause of this pattern is the increased frequency of gene exchange between different population germplasm resources [60].

The gene flow (*Nm*) is a critical factor influencing genetic differentiation among populations. Hamrick et al. suggested that when *Nm* > 4, the gene exchange between populations was relatively sufficient to counteract genetic drift and prevent genetic differentiation among populations [61]. In this study, the overall gene flow among provenances was 15.715, which aligns with the observed low level of genetic differentiation (*Fst* = 0.016). This high level of *Nm* has inhibited population differentiation and inbreeding, promoting individual variation and leading to a relatively uniform genetic background across populations. These findings are consistent with the results of the genetic structure analysis and variance analysis. Two primary factors may contribute to this pattern. First, it is related to the biological characteristics of *P. elliottii*. The species produces winged seeds and is wind-pollinated, with pollen-containing air sacs that allow it to travel relatively long distances [62,63]. Second, there has been frequent human-mediated exchange of germplasm materials between different populations over a long period, with most population materials being confined to Jiangxi Province [54]. This has resulted in low genetic diversity and a lack of significant genetic differentiation among populations. In the future selection of *P. elliottii* breeding materials, the focus should be on selecting and preserving superior individuals within each population. Additionally, selecting elite trees from major *P. elliottii* introduction areas could help expand the range of resource collection, enrich the diversity of genetic resources, and improve the breeding efficiency.

### 4.4. Genetic Structure and Clustering Analysis of P. elliottii Populations

Population structure analysis in plants primarily utilizes three different methods: STRUCTURE analysis, UPGMA clustering, and PCoA analysis. PCoA and UPGMA offer a visual representation of the distribution relationships among materials, while STRUCTURE analysis provides insights into the genetic background and gene flow within populations [64,65]. These three methods complement and corroborate each other, facilitating a comprehensive understanding of the genetic differences among materials. In this study, STRUCTURE analysis revealed that the 417 *P. elliottii* samples could be divided into two subgroups. Individuals from different sources clustered together within the same genetic structure, indicating that most samples had relatively homogeneous genetic backgrounds. Using a Q value threshold of 0.6 for classification, only seven samples had Q values below 0.6, suggesting that these samples have a more complex genetic structure and evidence of gene flow. Therefore, caution should be exercised when selecting these samples as breeding parents. Analysis using the UPGMA clustering also grouped the samples into two main clusters, and with few exceptions, the clustering patterns were consistent with the results of both the PCoA and STRUCTURE analyses.

## 5. Conclusions

This study examined the genetic diversity and population structure of 417 *P. elliottii* samples from three provenances using 19 polymorphic SSR markers. The results showed that *P. elliottii* exhibited moderate to high genetic diversity compared to other conifer species. The gene flow and genetic differentiation analyses indicated that the gene exchange among the provenances was relatively extensive, limiting genetic differentiation and resulting in low overall genetic differentiation. The molecular variance analysis revealed that most genetic variation occurred within populations. Therefore, breeding efforts should focus on selecting superior individuals from genetically diverse populations. The population structure analysis revealed that individuals from different sources were not strictly clustered according to their origins but were distributed across two subgroups, with seven samples classified into a mixed group, indicating complex genetic relationships. The presence of redundant genetic diversity among the 417 germplasm samples suggests that future work should aim to remove genetic redundancies. Overall, these findings provide valuable guidance for the efficient utilization of high-resin-yielding *P. elliottii* germplasm, the development of core germplasm, and the selection of hybrid parents for breeding new germplasm.

## Figures and Tables

**Table 1:** Information on the *P. elliottii* germplasm resources.

Provenance	Ways to Save	Source	Sample Number	Effective Amplification Number	Afforestation Site and Year
IP *	Introduced family trial forest	Georgia, Mississippi, and Florida, USA	113	112	Ji’an (1990)
		Florida, USA	30	8	Jingdezhen (2011)
		Florida, USA	10	10	Jingdezhen (2018)
		Kansas, USA	40	40	Ganzhou (2018)
FP	First-generation seed orchard	Early-introduced slash pine plantations in Jiangxi Province, China	44	44	Ji’an (1992)
			77	77	Xiajiang (1980)
IGP	Improved-generation seed orchard	Experimental forests planted with selected superior trees across Jiangxi province, China	47	47	Xiajiang (2015)
			79	79	Ji’an (2011)

* IP: Introduced family provenance; FP: First-cycle superior trees breeding provenance; IGP: Improved-generation superior trees breeding provenance, the same as below.

**Table 2:** Reaction system and SSR-PCR procedure for *P. elliottii*.

Reaction System		Reaction Procedure		
Component and Concentration	Volume (μL)	Cycling Parameters	Time (min, s)	
10 × buffer (plus Mg^2+^)	2	94 °C initial denaturation	5 min	30 cycles
10 mol/L dNTPs	1.6	94 °C denaturation	30 s	
forward primer	0.8	56–60 °C annealing	30 s	
reverse primer	0.8	72 °C extension	60 s	
Taq DNA polymerase (5 μ/µL)	0.1	72 °C final extension	7 min	
ddH_2_O	13.7	4 °C keep		
Template DNA (50 ng/μL)	1			

**Table 3:** Sequence information of 19 pairs of polymorphic EST-SSR primers.

Locus	Repeat Motif	Product Size (bp)	Primer Sequence (5′-3′)
Pe145169	(CT)11	257	F: TGAATCCTCGGAATTTCTGG
			R: TGCCATTGAAACAAGCTGAA
Pe144426	(AT)8	152	F: AATGCGAGTGGCAACAAAGT
			R: ATTTCACATTCCCGTTCTCG
Pe138370	(CCCTG)5	179	F: CCTGACGCAACATAATCCCT
			R: ATAAAAGACACACCCCGCAG
Pe130187	(TC)6	213	F: TTCTCATGCTAAGCACACGC
			R: ATTTCTTCCATGGGTTCGTG
Pe132622	(AC)10	262	F: TATAACGTCAGCCCAGGGAC
			R: TTGCTTCTGCAGGAAAGGTT
Pe146453	(CATT)5	257	F: CTGATCCACCCTCATCTGCT
			R: GGGAGCAACCAGAACAACAT
Pe134815	(CTG)7	181	F: CCCCAAACCCCAACTTAGAT
			R: AAGTGGGAAAAATGAGGGCT
Pe131259	(AT)7	183	F: GGATTGATCCAAGCCAACAG
			R: ACCCGGAGGCAAATCTATCT
Pe103802	(AT)7	141	F: GGATGATCAGGGCATGAAAT
			R: CATAAAAGTTGGCACCACCA
Pe123077	(AAT)6	129	F: ATTGGGTTGAATCCGAACAT
			R: CCAGACAAAATTGTGGCCTT
Pe106732	(AGG)5	163	F: CGGTGGAAGATTTAGGTCCA
			R: GAAAAACAGCGGCAGAAAAG
Pe145380	(AC)9	244	F: CAACATTTGCTGTGAGCGTT
			R: ATGCATCCCTGATGCTCTTC
Pe119033	(AT)6	267	F: TTCTTGATACATCGGGGCAT
			R: AAACCTGTTCAAATCCTCACAA
Pe135178	(TTC)5	145	F: ATTTCAGAAGGTCAATGCGG
			R: GCAGGACATAAATGGGCAGT
Pe140688	(GATG)5	264	F: ATGAACGCTTTAGTTCCCCC
			R: GTGATGCGAGATGTGCAGTT
Pe110222	(TTG)5	277	F: TCTGTAACTTGGACTGGCCC
			R: CAGCCACAGTAGGTGCAACA
Pe113019	(AG)7	240	F: ATCTAGCGATCCCGGAAGTT
			R: ACCACCTTCTTCCTCCCATT
Pe139538	(TTAG)5	280	F: TGAAAGGTGGAGATCCTTGG
			R: AGGTCTGAGAGCATGGAGGA
Pe114582	(TA)6	275	F: ATACCTAGGCAGATGCCCCT
			R: TTAGGCTGGACAACCCAAAC

**Table 4:** Transferability and percentage of the polymorphic loci for the tested SSR markers.

Sample	Amplification and Polymorphic (pair)	Amplification but No Polymorphism (pair)	No Amplification (pair)	Applicability (%)	Proportion of Polymorphic Sites (%)
*Pinus massoniana*	19	0	0	100%	100%
*Pinus taeda*	17	2	0	100%	89.47%

**Table 5:** Diversity parameters of 19 microsatellite loci in *P. elliottii*.

Locus	Na *	*Ne*	*I*	*Ho*	*He*	PIC
Pe145169	7	4.491	1.638	0.636	0.777	0.743
Pe144426	7	3.361	1.332	0.448	0.702	0.651
Pe138370	5	2.800	1.168	0.564	0.643	0.580
Pe130187	7	2.386	1.200	0.505	0.581	0.538
Pe132622	7	2.275	1.068	0.498	0.560	0.511
Pe146453	4	2.461	1.006	0.352	0.594	0.513
Pe134815	7	1.879	0.947	0.451	0.468	0.436
Pe131259	5	1.956	0.908	0.441	0.489	0.444
Pe103802	13	1.777	0.978	0.408	0.437	0.413
Pe123077	2	1.964	0.684	0.795	0.491	0.370
Pe106732	4	1.724	0.836	0.430	0.420	0.394
Pe145380	5	1.799	0.789	0.383	0.444	0.385
Pe119033	6	1.660	0.836	0.423	0.397	0.373
Pe135178	3	1.688	0.605	0.368	0.408	0.325
Pe140688	3	1.512	0.578	0.224	0.339	0.293
Pe110222	3	1.450	0.571	0.264	0.311	0.281
Pe113019	8	1.263	0.445	0.159	0.208	0.194
Pe139538	2	1.352	0.429	0.144	0.260	0.226
Pe114582	5	1.175	0.370	0.138	0.149	0.146
Mean	5.421	2.051	0.862	0.402	0.457	0.411

*: *Na*: number of alleles; *Ne*: number of effective alleles; *Ho*: observed heterozygosity; *He*: expected heterozygosity; *I*: Shannon’s information index; PIC: polymorphism information content, the same below.

**Table 6:** Polymorphism analysis of 19 pairs of primers in three provenances.

Locus	Provenance	*Na*	*Ne*	*I*	*Ho*	*He*	PIC
Pe146453	IP	4	2.475	0.997	0.357	0.596	0.519
	FP	4	2.195	0.866	0.422	0.544	0.440
	IGP	4	2.575	1.078	0.277	0.612	0.545
Pe145380	IP	3	1.647	0.657	0.389	0.393	0.335
	FP	4	1.844	0.775	0.314	0.458	0.390
	IGP	5	1.973	0.907	0.447	0.493	0.437
Pe145169	IP	7	4.273	1.565	0.645	0.766	0.728
	FP	7	3.858	1.475	0.708	0.741	0.695
	IGP	7	5.125	1.765	0.553	0.805	0.779
Pe144426	IP	5	3.270	1.271	0.518	0.694	0.637
	FP	6	3.189	1.312	0.298	0.686	0.635
	IGP	5	3.518	1.373	0.500	0.716	0.668
Pe140688	IP	2	1.302	0.394	0.161	0.232	0.205
	FP	3	1.514	0.625	0.314	0.340	0.309
	IGP	3	1.780	0.662	0.222	0.438	0.349
Pe132622	IP	5	2.158	0.968	0.453	0.537	0.484
	FP	6	2.130	1.006	0.513	0.531	0.479
	IGP	6	2.573	1.181	0.544	0.611	0.564
Pe131259	IP	4	1.781	0.787	0.455	0.438	0.395
	FP	5	1.928	0.903	0.525	0.481	0.432
	IGP	4	2.177	0.974	0.344	0.541	0.488
Pe130187	IP	7	1.998	1.022	0.518	0.500	0.467
	FP	7	2.606	1.259	0.525	0.616	0.572
	IGP	7	2.646	1.259	0.468	0.622	0.566
Pe123077	IP	2	1.867	0.657	0.708	0.464	0.357
	FP	2	1.976	0.687	0.891	0.494	0.372
	IGP	2	2.00	0.693	0.815	0.500	0.375
Pe119033	IP	5	1.444	0.588	0.343	0.308	0.279
	FP	5	1.610	0.794	0.376	0.379	0.359
	IGP	6	2.045	1.048	0.571	0.511	0.480
Pe114582	IP	4	1.116	0.261	0.108	0.104	0.102
	FP	4	1.193	0.363	0.174	0.162	0.155
	IGP	5	1.241	0.467	0.142	0.194	0.189
Pe139538	IP	2	1.079	0.162	0.076	0.074	0.071
	FP	2	1.371	0.442	0.223	0.270	0.234
	IGP	2	1.734	0.614	0.160	0.423	0.334
Pe138370	IP	4	2.501	1.051	0.575	0.600	0.526
	FP	4	3.263	1.268	0.583	0.694	0.638
	IGP	5	2.736	1.165	0.532	0.634	0.572
Pe135178	IP	3	1.611	0.582	0.327	0.379	0.310
	FP	2	1.642	0.580	0.400	0.391	0.315
	IGP	2	1.823	0.644	0.393	0.451	0.350
Pe134815	IP	6	1.804	0.872	0.464	0.446	0.408
	FP	4	1.790	0.853	0.455	0.441	0.409
	IGP	7	2.056	1.060	0.432	0.514	0.481
Pe113019	IP	4	1.160	0.297	0.124	0.138	0.131
	FP	3	1.320	0.425	0.116	0.243	0.215
	IGP	8	1.356	0.594	0.246	0.262	0.248
Pe110222	IP	2	1.209	0.315	0.117	0.173	0.158
	FP	3	1.751	0.726	0.451	0.429	0.370
	IGP	3	1.533	0.647	0.286	0.348	0.319
Pe106732	IP	4	1.565	0.683	0.391	0.361	0.326
	FP	4	1.403	0.596	0.275	0.287	0.271
	IGP	4	2.351	1.092	0.632	0.575	0.535
Pe103802	IP	9	1.545	0.809	0.329	0.353	0.338
	FP	8	1.973	0.979	0.438	0.493	0.449
	IGP	10	1.918	1.083	0.484	0.479	0.456
Mean	IP	4.316	1.885	0.734	0.371	0.398	0.356
	FP	4.368	2.029	0.839	0.421	0.457	0.407
	IGP	5.000	2.272	0.964	0.424	0.512	0.460
Total	-	4.561	2.062	0.845	0.405	0.456	0.408

**Table 7:** Genetic differentiation and gene flow in the provenances of *P. elliottii*.

Locus	Fis *	*Fit*	*Fst*	*Nm*	Locus	*Fis*	*Fit*	*Fst*	*Nm*
Pe145169	0.175	0.183	0.009	26.595	106732	−0.061	−0.019	0.039	6.123
Pe144426	0.373	0.376	0.006	43.219	145380	0.144	0.148	0.005	53.260
Pe138370	0.124	0.131	0.008	30.356	119033	−0.078	−0.060	0.017	14.541
Pe130187	0.131	0.145	0.017	14.397	135178	0.083	0.090	0.008	30.815
Pe132622	0.101	0.106	0.006	45.202	140688	0.310	0.335	0.037	6.550
Pe146453	0.397	0.406	0.015	16.605	110222	0.100	0.129	0.032	7.517
Pe134815	0.036	0.042	0.006	40.009	113019	0.244	0.252	0.010	25.888
Pe131259	0.094	0.107	0.014	17.531	139538	0.401	0.451	0.084	2.710
Pe103802	0.055	0.067	0.012	20.022	114582	0.079	0.086	0.007	34.196
Pe123077	−0.655	−0.634	0.013	19.125	Mean	0.110	0.124	0.016	15.715

* *Fis*: inbreeding coefficient at the population level; *Fit*: inbreeding coefficient at total populations; *Fst*: proportion of differentiation among populations, the same below.

**Table 8:** Analysis of the molecular variance (AMOVA) of *P. elliottii*.

Source	Degrees of Freedom	Sum of Squares	Variance Components	Percent of Variation (%)
Among provenance	2	54.576	0.085	2.10%
Within provenance	831	3289.002	3.958	97.90%
Total	833	3343.578	4.043	100%

**Table 9:** Genetic distance (lowerleft) and genetic similarity (upperright) between three provenances.

Provenance	IP	FP	IGP
IP	****	0.986	0.980
FP	0.014	****	0.981
IGP	0.020	0.019	****

**Table 10:** Statistics of the population structure for 417 *P. elliottii*.

Provenance	Ways to Save	Subgroup I	Subgroup II	Mix Group
IP	Introduced family trial forest	159	4	7
FP	First-generation seed orchard	105	16	0
IGP	Advanced-generation seed orchard	88	38	0

## Data Availability

Data are contained within the article.
